# The Fundamental Role of Nutrients for Metabolic Balance and Epigenome Integrity Maintenance

**DOI:** 10.3390/epigenomes9030023

**Published:** 2025-07-09

**Authors:** Ana Paula de Souza, Vitor Marinho, Marcelo Rocha Marques

**Affiliations:** Department of Bioscience, Division of Histology and Embryology, State University of Campinas UNICAMP, Campinas 13414-903, Brazil

**Keywords:** diet, epigenetic, DNA methylation, histone modification, metabolism, TCA cycle, one-carbon cycle

## Abstract

Epigenetic modifications act as crucial regulators of gene activity and are influenced by both internal and external environmental factors, with diet being the most impactful external factor. On the other hand, cellular metabolism encompasses a complex network of biochemical reactions essential for maintaining cellular function, and it impacts every cellular process. Many metabolic cofactors are critical for the activity of chromatin-modifying enzymes, influencing methylation and the global acetylation status of the epigenome. For instance, dietary nutrients, particularly those involved in one-carbon metabolism (e.g., folate, vitamins B12 and B6, riboflavin, methionine, choline, and betaine), take part in the generation of S-adenosylmethionine (SAM), which represents the main methyl donor for DNA and histone methylation; α-ketoglutarate and ascorbic acid (vitamin C) act, respectively, as a co-substrate and cofactor for Ten-eleven Translocation (TET), which is responsible for DNA demethylation; and metabolites such as Acetyl-CoA directly impact histone acetylation, linking metabolism of the TCA cycle to epigenetic regulation. Further, bioactive compounds, such as polyphenols, modulate epigenetic patterns by affecting methylation processes or targeting epigenetic enzymes. Since diet and nutrition play a critical role in shaping epigenome functions and supporting human health, this review offers a comprehensive update on recent advancements in metabolism, epigenetics, and nutrition, providing insights into how nutrients contribute to metabolic balance, epigenome integrity maintenance and, consequently, disease prevention.

## 1. Introduction

Epigenetics refers to mitotically heritable changes in gene expression that result from chromatin structure modifications without altering the underlying DNA sequence [1,2]. The primary epigenetic modifications include cytosine methylation within CpG dinucleotides, particularly in CpG islands located in genomic regulatory elements; post-translational modifications of histone protein tails; and the regulatory roles of non-coding RNAs. Also, posttranscriptional modifications of RNA, known as the “epitranscriptome,” such as modifications of N6-methyladenosine (m6A), influence cell biology by regulating mRNA processing, translation, and stability [1,3,4]. Given that DNA methylation and various post-translational histone modifications and also posttranscriptional modifications of RNA are regulated by enzymes that function as readers, writers, and erasers of these chemical marks on chromatin [1,3,5]—and these enzymes rely on metabolites as substrates or cofactors for a wide range of epigenetic reactions [2,6,7]—it is evident that metabolic pathways influence the epigenome. On the other hand, the epigenome impacts cellular metabolism on a global scale by regulating genetic transcription [7]. So, the nutrients found in our diet impact both cellular metabolism and the epigenome.

The concept of an “optimized epigenetic diet” was introduced relatively recently (2011) based on the premise that various nutrients and bioactive food components can modulate epigenetic processes, thus contributing to the promotion of human health [8]. In fact, several chemicals found in supplements or natural foods have the potential to interact with epigenetic factors [9]; regardless of whether a diet is considered “regular” or specifically “epigenetic”, any kind of diet has the potential to impact the cellular epigenetic pattern [10,11]. The challenge lies in measuring how the bioavailability of a single chemical compound differs from its bioavailability within the complex matrix of a natural food, how these chemical substances interact, and how the myriad of possible combinations of the chemical modifications of all histone tails, in association with DNA methylation/demethylation status, and the action of numerous ncRNAs contribute to generating the functional epigenome of each distinct cell type.

Changes in the concentrations of specific metabolites are believed to serve as signaling cues that dynamically regulate gene expression by influencing chromatin structure and function [12,13]. Thereby, the quantity and quality of nutrients in the diet play a critical role in modulating DNA methylation, as well as the methylation, acetylation, and phosphorylation of proteins since central metabolites, acting as substrates or allosteric cofactors, rapidly influence the epigenome by fine-tuning the activity of epigenetic enzymes. Dietary nutrients, particularly those involved in one-carbon metabolism—such as folate, vitamin B12, vitamin B6, riboflavin, methionine, choline, and betaine—affect DNA methylation by regulating the levels of the universal methyl donor S-adenosylmethionine (SAM) [6,14].

DNA methylation, the addition of a methyl group to cytosine bases, is a well-established epigenetic mark that can alter chromatin structure and gene expression without changes to the underlying DNA sequence. Aberrant DNA methylation patterns are implicated in various pathological states, including cancer, and aging. The nutrients highlighted are integral to the one-carbon metabolism pathway, which is central to the synthesis of SAM, which functions as the principal methyl donor for DNA methyltransferases (DNMTs), the enzymes responsible for catalyzing DNA methylation. Therefore, dietary intake of these nutrients directly impacts the availability of methyl groups and, consequently, the landscape of DNA methylation across the genome—the epigenomic profile. Compelling evidence demonstrated that a methyl-deficient diet (lacking sufficient folate, choline, and methionine) induced abnormal DNA methylation in the livers of mice, leading to the development of methyl-deficiency-induced hepatocarcinoma [15]. This is because the availability of methyl donors, directly dependent on the aforementioned nutrients, dictates the activity of DNMTs (read more in Section 3).

Additionally, some vitamins, notably ascorbic acid (vitamin C or ascorbate), serve as essential cofactors for enzymes such as Ten-eleven Translocation (TET) and Jumonji C-domain-containing histone demethylases (JHDM). These enzymes, which belong to the Fe(II)- and α-ketoglutarate-dependent dioxygenase superfamily [6,16], are common to DNA methylation and histone modification processes, respectively, further influencing gene expression. Metabolic production of acetyl coenzyme A (acetyl-CoA) is also directly linked to histone acetylation, highlighting the connection between metabolism and epigenetic regulation [6]. Furthermore, bioactive compounds like polyphenols can modulate epigenetic patterns by influencing methylation processes or directly targeting enzymes involved in DNA methylation and histone modifications [17,18,19].

Given the critical role of diet and nutrition in shaping epigenome functions and supporting human health, this review provides a comprehensive update on recent advancements in metabolism, epigenetics, and nutrition, offering insights into how nutrients contribute to health and disease prevention.

## 2. One-Carbon Metabolism and Methyl Groups

The nutritional connection to DNA and histone methylation lies in one-carbon metabolism, which encompasses the methionine cycle (including methionine remethylation and the transsulfuration pathway) and the folate cycle. These critical pathways depend on dietary nutrients such as folate, serine, glycine, vitamins B2, B6, and B12, methionine, and choline (betaine), which serve as essential substrates or cofactors to maintain the homeostasis of both cycles. S-adenosylmethionine (SAM), a universal methyl donor, is derived from the folate cycle [2,20,21]. Specifically, folic acid is converted into 5,10-methylenetetrahydrofolate (5,10-MTHF), which participates in two key processes. In the cytosol, 5,10-MTHF donates methyl groups for the synthesis of thymine nucleotides in DNA, converting deoxyuridine 5′-monophosphate (dUMP) to deoxythymidine 5′-monophosphate (dTMP) [14,20,22].

Additionally, 5,10-MTHF is reduced to 5-methyltetrahydrofolate (5-MTHF), which serves as a methyl donor for the recycling of homocysteine (HCY) into methionine [23]. This process, a vitamin B12-dependent reaction, forms a feedback system that balances the methionine cycle and generates SAM, an essential factor for methylation reactions which serves as the primary methyl donor utilized by DNA methyltransferases (DNMT-1, DNMT-3A, DNMT-3B), histone lysine N-methyltransferases and histone arginine N-methyltransferases, and methyltransferase-like (METTL3 and METTL14), which act specific to DNA, histone, and RNA, respectively [2,14,23]. During methylation processes, SAM is converted into S-adenosylhomocysteine (SAH), which is subsequently hydrolyzed to homocysteine (HCY). Under optimal methionine and folate cycle conditions, elevated levels of S-adenosylmethionine (SAM) promote homocysteine (HCY) degradation through the two-step transsulfuration pathway, which requires vitamin B6 as a cofactor. Additionally, SAM acts as an allosteric inhibitor of methylenetetrahydrofolate reductase (MTHFR) [24] and an activator of cystathionine β-synthase (CBS), the enzyme responsible for catalyzing the condensation of serine with HCY to form cystathionine. Cystathionine is subsequently cleaved to generate cysteine, a precursor for glutathione—an essential cellular redox regulator—as well as propionyl-CoA, which work as a substrate for mitochondrial metabolic pathways [14,20,22,25]. Consequently, one-carbon metabolism is intricately linked to cellular redox balance, mitochondrial function, and energy metabolism.

Disruptions in the one-carbon cycle can affect the balance of the SAM:SAH ratio, thereby influencing the availability of methyl groups for nucleic acid and protein methylation and potentially leading to increased homocysteine production [26]. Hyperhomocysteinemia has been associated with a risk of cardiovascular and neurodegenerative diseases. Homocysteine levels are tightly controlled through two key pathways: remethylation back to methionine and irreversible breakdown via transsulfuration. Remethylation is a crucial step for the methyl cycle and proceeds through two distinct routes. The first relies on folate and vitamin B12. Here, 5-methyl-THF (a folate coenzyme) transfers a methyl group to homocysteine, a reaction catalyzed by the vitamin B12-dependent enzyme methionine synthase (MS) [27].

Adequate folate and/or B12 are therefore essential for maintaining homocysteine balance within cells and the circulatory system. The availability of 5-methyl-THF itself depends on the reduction of 5,10-methylene-THF by the enzyme MTHFR. A common variant in the MTHFR gene (C677T) can impair this reduction, resulting in elevated homocysteine levels [14]. The second remethylation pathway, primarily active in the liver and kidneys, bypasses the need for folate and instead uses betaine (derived from choline oxidation) as a methyl donor, catalyzed by BHMT. While this betaine-dependent pathway is concentrated in the liver and kidneys, the folate/B12-dependent route operates in all tissues.

## 3. The Nutritional Emphasis on MTHFR Gene Variants and Differentially Methylated Regions

As previously mentioned, MTHFR is a key enzyme in the methylation cycle, catalyzing the conversion of 5,10-methylenetetrahydrofolate to 5-methyltetrahydrofolate—a crucial co-substrate for the remethylation of homocysteine to methionine and the regeneration of tetrahydrofolate. Reduced MTHFR activity (~70%) is commonly observed in individuals carrying genetic variants of the *MTHFR* gene, particularly at position 677 (rs1801133), where a substitution of cytosine (C, wild type) to thymine (T, variant) results in an alanine-to-valine change at amino acid 222 (Ala222Val) in exon 4, leading to a thermolabile and functionally impaired enzyme. Similarly, a variant at position 1298 (rs1801131) involves an adenine (A, wild type) to cytosine (C, variant) substitution, causing a glutamic acid to alanine change at amino acid 429 (Glu429Ala) in exon 7 [14,28].

The *MTHFR* 677C>T polymorphism is present in approximately 25% of the global population, with the highest frequency observed in Hispanics (47%), followed by Europeans (36%), East Asians (30%), South Asians (12%), and Africans (9%), according to the 1000 Genomes Project. Among Europeans, approximately 13.5% are homozygous for the variant allele. Similarly, the *MTHFR* 1298A>C polymorphism is found in about 25% of the global population, with the highest prevalence in Southeast Asians (42%) and Europeans (31%). In contrast, its frequency among Hispanics and Africans is around 15%. Notably, approximately 11% of Europeans are homozygous for the 1298A>C variant allele. While the frequencies of individual polymorphisms at the *MTHFR* 677 and 1298 loci have been extensively studied, the distribution of their combined configurations, including diplotypes and haplotypes, remains less well characterized—particularly across different ethnic groups. Additionally, the genetic impact of these variant combinations on enzyme function has not been fully elucidated [29,30].

A logical inference, based on the functional impact of genetic variations in the *MTHFR* gene that reduce enzyme activity, coupled with additional SNPs that downregulate gene expression, suggests that these variations could result in diminished SAM levels and, consequently, a reduction in DNA methylation (global hypomethylation). However, the underlying relationship is far more intricate than it initially appears. A recent comprehensive literature review examining studies from the past 15 years uncovered a spectrum of conflicting, and at times contradictory, results [14]. As anticipated, a correlation between homozygosity for the rs1801133 TT genotype and global hypomethylation was identified in the blood cells of Russian individuals [31]. Additionally, the presence of the T allele was linked to a reduced hypomethylation of LINE-1 sequences in individuals across various clinical contexts [32,33].

In contrast, a study involving a Brazilian cohort of mothers of children with Down syndrome and mothers of children without Down syndrome revealed no association between folic acid levels and DNA methylation associated with rs1801133 or rs1801131 genotypes [34]. Numerous other studies have found no association between genetic variations in the MTHFR gene and DNA hypomethylation across a range of diseases. Furthermore, some studies have even reported an association between these variants with DNA hypermethylation. These findings indicate that the presence or absence of MTHFR risk alleles (rs1801133 or rs1801131) cannot be straightforwardly correlated with the DNA methylation status [14].

## 4. Metabolism and Its Connection to DNA, RNA, and Histone Methylation

The CpG dinucleotide methylation in DNA is the most extensively studied epigenetic event, and it can dramatically impact gene expression, especially when its occurrence is associated with regions harboring regulatory elements of the genome such as enhancers and gene promoters. However, epigenetic modifications can potentially interfere with transcriptional regulation and subsequently with protein synthesis and are not limited to DNA. There is a wide range of post-translational modifications that occur in histone proteins and regulate chromatin accessibility, as well as chemical modifications that occur in messenger RNA molecules and impact the subsequent step of protein translation.

It is crucial to understand that all epigenetic modifications, whether in DNA, histones, or RNA, result from the activity of specific enzymes that methylate or demethylate DNA, histones, and RNA, or acetylate, deacetylate, phosphorylate, and ubiquitinate histone proteins [2]. Furthermore, elements derived from cellular energy metabolism serve as substrates, co-substrates, and cofactors for a wide range of these enzymes.

DNA methyltransferases (DNMT1, DNMT3A, DNMT3B) are responsible for the methylation of cytosine bases in DNA, leading to the formation of 5-methylcytosine, known as the fifth base of DNA. This epigenetic mark is strongly associated with the repression or complete silencing of gene transcription in many cells of eukaryotic organisms. The activity of the DNMT1 enzyme is associated with the maintenance of the cellular genome methylation pattern throughout mitotic and meiotic cell divisions, and thus, with the stability of the differentiated cellular phenotype as well as genomic imprinting in germ cells. In contrast, the activity of DNMT3A and 3B enzymes is associated with the gain of methylation at originally unmethylated sites in the genome [35,36].

Histone proteins are methylated by enzymes known as histone methyltransferases (HMTs) that catalyze the addition of one (mono-), two (di-), or three (tri-) methyl groups to lysine and arginine residues of histone, particularly in the tails of histones 3 (H3) and 4 (H4). Consequently, this generates a state of higher or lower chromatin compaction depending on the exact position of the lysine in H3 or H4, containing several types of HMT enzymes [2,6,37]. For instance, the enzyme EZH2 represents an important HMT that is responsible for transferring tri-methyl groups to the lysine amino acid located at position 27 of the H3 tail, creating the epigenetic mark H3K27me3, which is strongly associated with chromatin repression and silencing of gene transcription when present in regulatory regions [38]. On the other hand, tri-methylation of lysine 4 of H3 tail is a permissive epigenetic mark of gene transcription, and it is associated with the functional activity of SET1/MLL enzymes [39].

Both DNMT and HMT enzymes utilize S-adenosylmethionine (SAM) generated within the one-carbon cycle as the principal methyl group donor [14,25]. Elevated concentrations of SAM function as an allosteric inhibitor of the methylenetetrahydrofolate reductase (MTHFR) enzyme, whereas increased levels of SAH can competitively inhibit the activity of DNMTs since the chemical structure of SAH is very similar to that of SAM, competing with SAM for the same active site on the enzyme. Hence, it is important to ensure adequate intake of B-complex vitamins, particularly B12, B6, and B9, as they serve as essential cofactors within the one-carbon metabolism cycle, according to what is described in Section 2. Additionally, consideration of genetic variants in the *MTHFR* gene is recommended, especially in populations with a higher prevalence of the risk allele associated with the rs1801133 variant [14].

The precise equilibrium of cytosines and 5-methylcytosines within the genome is meticulously regulated through a dynamic interplay between cytosine methylation, catalyzed by DNA methyltransferases (DNMTs), and demethylation, orchestrated by the Ten-eleven Translocation (TET) enzyme family. TET enzymes comprise a class of Fe(II)- and 2-oxoglutarate-dependent dioxygenases that catalyze the stepwise oxidation of 5-methylcytosine, initiating the conversion to 5-hydroxymethylcytosine [40,41]. This oxidative modification facilitates passive demethylation, as hydroxymethylated cytosines—and their further oxidized derivatives, 5-formylcytosine and 5-carboxylcytosine—are recognized and processed by the nuclear base excision repair machinery [6]. Through this mechanism, modified cytosines are replaced with unmodified cytosines, thus preserving the dynamic epigenetic landscape essential for regulating gene expression and maintaining genomic integrity.

Once more a critical interplay between nutrition and metabolism emerges. The enzymatic activity of TET enzymes is optimized by vitamin C, which is a crucial co-factor since it prevents the premature oxidation of iron in TET enzymes, maintaining iron in its reduced form (Fe(II)), which is more efficient for catalyzing the reaction. Without vitamin C, Fe(II) could be oxidized to Fe(III), which would inactivate the TET enzymes and prevent the modification of 5-methylcytosine. In a manner analogous to TET enzymes, vitamin C (ascorbic acid) functions as a critical cofactor for Jumonji C-domain-containing histone demethylases (JHDM)**,** a family of dioxygenases that catalyze the removal of methyl groups from histones, specifically from methylated lysine residues [6].

Adenosine methylation in RNA, particularly the m6A (N6-methyladenosine) modification, stands as one of the most extensively studied and pivotal epitranscriptomic modifications, playing a central role in the regulation of post-transcriptional gene expression [42]. The m6A modification is catalyzed by a complex of methyltransferase-like enzymes, primarily METTL3 and METTL14, which work together to add a methyl group to the nitrogen at position 6 of adenosine within specific sequence motifs on messenger RNA (mRNA). This modification is highly dynamic and occurs predominantly in the 3′ untranslated regions (UTR), exonic regions, and introns of mRNA, influencing various aspects of RNA metabolism [4,43].

The presence of m6A serves as a key regulator of multiple cellular processes, including RNA processing, where it modulates splicing, capping, and editing, as well as nuclear export and transport [4,44]. Furthermore, m6A impacts mRNA stability, where it can promote decay or enhance the persistence of mRNA in the cytoplasm. In addition, translation efficiency is tightly regulated by m6A, as it can facilitate or inhibit ribosome loading depending on the context and associated binding proteins. Through these mechanisms, m6A orchestrates a fine-tuned network of regulatory events, which ensures the precise control of gene expression in response to developmental cues, environmental stress, and various cellular signals. The reversible nature of m6A, involving both methylation and demethylation processes, further highlights its role in maintaining cellular plasticity and homeostasis [4]

The demethylation of m6A in RNA is a dynamic and reversible process mediated by a specialized family of RNA demethylases. The fat mass and obesity-associated protein (FTO) was the first demethylase identified to catalyze the removal of the m6A modification, followed by ALKBH5 (AlkB homolog 5), which also contributes to this process. These demethylases operate through an oxygen- and 2-oxoglutarate-dependent mechanism, utilizing these cofactors to oxidize the methyl group attached to the nitrogen 6 (N6) position of the adenosine base [45]. This action results in the removal of the methyl group and the reversion of the m6A modification to the original adenosine. Both FTO and ALKBH5 have been shown to exhibit specificities for m6A-containing RNA, influencing various cellular processes such as RNA stability, splicing, translation efficiency, and transport [2,45]. These enzymes are part of a broader regulatory network that ensures the reversible nature of RNA modifications, allowing for the fine-tuned regulation of gene expression in response to cellular needs and environmental cues. Importantly, the demethylation of m6A is not merely a passive process, but it is an active modulator of epitranscriptomic regulation, affecting key biological processes like development, cell differentiation, and response to stress.

## 5. Importance of TCA Cycle and Generation of Acetyl Groups to Maintenance of Epigenetic Landscape

The TCA cycle, also known as the Krebs cycle, is a critical metabolic pathway that occurs within the mitochondrial matrix. Here, Acetyl-Coenzyme A (Acetyl-CoA), which consists of an acetyl group covalently attached to coenzyme A, functions as a central metabolic intermediate [6]. Coenzyme A itself is a complex derivative synthesized from vitamin B5 (pantothenate) and cysteine, with multiple steps in its biosynthesis pathway critically requiring the input of ATP. Acetyl-CoA integrates various biochemical pathways and undergoes oxidative catabolism to generate ATP, the primary energy currency of the cell. Derived from pyruvate following glycolysis, Acetyl-CoA enters the citric acid cycle to drive cellular energy production [46]. In addition to its pivotal role in metabolism, Acetyl-CoA also acts as a key regulator of gene expression through its involvement in histone acetylation [6].

Acetyl-CoA is distributed across distinct cellular compartments, including the mitochondria and the nuclear–cytosolic region. The inner mitochondrial membrane is impermeable to Acetyl-CoA due to its highly charged nature. In contrast, the nuclear–cytosolic exchange of Acetyl-CoA is more nuanced; while nuclear pores allow for some diffusion, the concentration and availability of nuclear Acetyl-CoA are also significantly influenced by in situ synthesis within the nucleus, for instance, via enzymes like nuclear ATP citrate lyase (ACLY) that generate Acetyl-CoA from transported precursors. Within the nucleus, during histone acetylation, acetyl groups from Acetyl-CoA are transferred to lysine residues on the N-terminal tails of histones by histone acetyltransferases (HATs) [6,47]. This post-translational modification neutralizes the positive charge of histones, diminishing their interaction with the negatively charged DNA. Consequently, the chromatin structure becomes more relaxed, enhancing DNA accessibility to the transcriptional machinery and promoting gene activation [48].

The process of histone acetylation is highly dynamic and reversible, relying on a delicate equilibrium between HATs and histone deacetylases (HDACs) [49]. Any disruption in this balance (whether due to excess or deficiency) can significantly impact gene expression, contributing to the development of numerous physiological conditions and diseases, and Acetyl-CoA takes part in this balance since it acts as the main acetyl donor. For instance, **a** diet rich in fat can disrupt Acetyl-CoA metabolism in a deleterious manner. On the other hand, a recent study demonstrated that the generation of Acetyl-CoA via specific metabolites facilitates cardiac repair following myocardial infarction through histone acetylation [50]. Additionally, research indicates that the biosynthesis of Acetyl-CoA may influence acquired resistance to HAT inhibitors, particularly those targeting the CBP/p300 proteins [51].

The regulation of HAT activity is intimately linked to Acetyl-CoA metabolism. Another study revealed that reduced expression of Acetyl-CoA carboxylase (ACC1) leads to an increase in histone acetylation, highlighting the connection between lipid biosynthesis and epigenetic regulation [52]. These insights emphasize the complex interdependence between Acetyl-CoA metabolism and the epigenetic control of gene expression, suggesting that variations in Acetyl-CoA levels can profoundly influence cellular processes through the modulation of HAT activity.

## 6. The Importance of Safeguarding Mitochondrial Metabolism and the Epigenome Against Oxidative Damage

Oxidative stress profoundly disrupts key metabolic processes, including the citric acid cycle, Acetyl-CoA synthesis, mitochondrial function, and lipid oxidation. Oxidative stress arises from an imbalance between the generation of reactive oxygen species (ROS) and the cell’s capacity to neutralize them, culminating in cellular damage. ROS, such as hydroxyl radicals (OH•) and hydrogen peroxide (H_2_O_2_), can inflict significant damage on mitochondrial membranes, including the inner mitochondrial membrane. This membrane is crucial for energy production and for regulating the passage of critical molecules. While the inner membrane itself is impermeable to Acetyl-CoA, it plays a vital role in the indirect transport of its precursors and metabolic products, such as pyruvate, fatty acids, and citrate, thereby influencing mitochondrial metabolism [53].

Excessive ROS lead lipid oxidation within mitochondria, disrupting the conversion of fatty acids into Acetyl-CoA via β-oxidation. Acetyl-CoA plays a pivotal role in the citric acid cycle, and any disruption in its synthesis leads to a marked reduction in ATP production, thus impairing cellular energy homeostasis. Oxidative stress can also directly compromise the functionality of enzymes central to the citric acid cycle, including citrate synthase, isocitrate dehydrogenase, and alpha-ketoglutarate dehydrogenase. This damage diminishes the cycle’s efficiency, resulting in a reduced generation of NADH and FADH_2_—vital electron carriers that fuel the electron transport chain and facilitate ATP synthesis [6].

Sirtuins constitute a family of NAD^+^-dependent deacetylases (HDAC) which are integral to the regulation of cellular metabolism, aging, and the response to stress [54]. These enzymes utilize NAD^+^ as an essential cofactor to catalyze the removal of acetyl groups from target proteins, thereby influencing a myriad of critical metabolic pathways [6,54,55]. Research has demonstrated a profound relationship between the activity of sirtuins and intracellular NAD^+^ levels. A study performed more than a decade ago elucidated that diminished NAD^+^ concentrations were concomitant with reduced SIRT1 activity in various organs of aged Wistar rats, including the liver, heart, kidney, and lungs [56]. This decline in NAD^+^ levels was associated with heightened oxidative stress and DNA damage, underscoring the pivotal role of NAD^+^ in maintaining sirtuin function and safeguarding against cellular senescence [57]. Importantly, the intracellular NAD^+^/NADH ratio also plays a critical role in regulating sirtuin activity, as a more oxidized cellular environment (higher NAD^+^ relative to NADH) favors sirtuin-mediated deacetylation, whereas an imbalance toward NADH may inhibit their function. Moreover, the modulation of NAD^+^ levels has garnered attention as a potential therapeutic avenue for aging-related pathologies. For instance, the supplementation of NAD^+^ precursors such as nicotinamide mononucleotide (NMN) has exhibited promising results in preclinical models, with evidence suggesting the restoration of sirtuin activity and subsequent improvements in metabolic health. However, it is important to acknowledge that, while these initial findings are encouraging, the clinical efficacy of such interventions is still under investigation in ongoing trials. Therefore, NAD^+^ serves as an indispensable cofactor for sirtuins, and the maintenance of optimal NAD^+^ levels is crucial for the proper functioning of these enzymes and the overall integrity of cellular processes. Dysregulation of NAD^+^ levels can significantly impair sirtuin activity, contributing to the onset of aging-associated disorders and pathological conditions.

In summary, oxidative damage can modulate the activity of histone acetyltransferases (HATs), thereby disrupting epigenetic regulation of gene expression. This interference can significantly affect cellular responses to oxidative stress and compromise the cell’s ability to repair damage, further exacerbating the overall impact on cellular function and integrity. Thus, a diet rich in antioxidant foods is strategically essential for maintaining the balance of mitochondrial function, metabolic biochemical pathways, and the epigenetic landscape.

## 7. Polyphenols: Powerful Antioxidants and Key Epigenetic Regulators for Health

Polyphenols represent a diverse class of bioactive phytochemicals abundantly found in plant-based foods, including berries, cruciferous and leafy vegetables, teas, and red wine. A growing body of scientific evidence underscores their pivotal role in epigenetic regulation and the promotion of longevity and healthy aging. These compounds exert profound modulatory effects on epigenetic mechanisms, particularly through alterations in DNA methylation landscapes, post-translational histone modifications (e.g., acetylation, methylation, and phosphorylation), and the regulation of non-coding RNAs [58,59]. Such epigenetic remodeling plays a critical role in orchestrating the transcriptional activity of genes implicated in chronic inflammation, oxidative stress, and cellular senescence—hallmarks of aging and age-related pathologies, including cardiovascular disease, neurodegenerative disorders, and cancer.

These bioactive phytochemicals are broadly classified into polyphenol, flavonoid, and non-flavonoid subclasses, each possessing distinct biochemical properties and molecular targets. Flavonoid polyphenols—such as quercetin, kaempferol, epigallocatechin-3-gallate (EGCG), luteolin, apigenin, silybin, hesperetin, and genistein—have been extensively investigated for their regulatory effects on key longevity-associated signaling pathways, including the Nrf2/ARE antioxidant response, NF-κB-mediated inflammation, and sirtuin-dependent metabolic homeostasis [60,61].

EGCG, the predominant catechin in green tea, exhibits a profound influence on the mechanistic target of rapamycin (mTOR) pathway, a central regulator of cellular proliferation, growth, and autophagy [62]. By suppressing mTOR signaling, EGCG enhances autophagic flux, mitigates proteotoxic stress, and reinforces cellular resilience to metabolic and environmental insults—factors intimately linked to lifespan extension. Furthermore, EGCG acts as a potent activator of AMP-activated protein kinase (AMPK), a crucial metabolic sensor that promotes mitochondrial biogenesis, enhances lipid metabolism, and exerts anti-inflammatory effects [62,63,64].

Quercetin, another well-characterized flavonoid, similarly modulates these longevity-associated pathways by stimulating AMPK activation, thereby reinforcing energy homeostasis and cellular stress resistance. Notably, both EGCG and quercetin have been identified as sirtuin activators, particularly influencing SIRT1 and SIRT3, which are instrumental in mitochondrial function, genomic stability, and metabolic adaptation [65]. These interactions underscore the capacity of flavonoid polyphenols to engage in intricate crosstalk between cellular energy sensing and epigenetic regulation, reinforcing their potential as therapeutic agents in mitigating age-related decline.

Among non-flavonoid polyphenols, resveratrol, curcumin, sulforaphane isothiocyanate, phenethyl isothiocyanate, and allyl mercaptan exhibit potent epigenetic effects, particularly through the activation of sirtuins, inhibition of histone deacetylases (HDACs), and modulation of DNA methyltransferases (DNMTs). Resveratrol, in particular, has garnered considerable attention for its regulatory influence on histone acetyltransferase p300/CBP, a key co-activator involved in chromatin remodeling and transcriptional control [66,67]. By modulating p300 activity, resveratrol can fine-tune the acetylation status of pivotal transcription factors, including those of the Forkhead box O (FOXO) family—master regulators of stress resilience, metabolism, and longevity [67].

Resveratrol has also been shown to enhance FOXO-driven transcription of genes encoding antioxidant enzymes (e.g., catalase, superoxide dismutase) and DNA repair factors while concomitantly repressing genes associated with apoptosis and cellular senescence [59]. Moreover, resveratrol’s interaction with sirtuins, particularly SIRT1, facilitates FOXO deacetylation, modulating its transcriptional dynamics in response to metabolic and oxidative stress. These synergistic mechanisms collectively contribute to resveratrol’s well-documented effects on lifespan extension and metabolic resilience across various model organisms.

**Figure 1:**
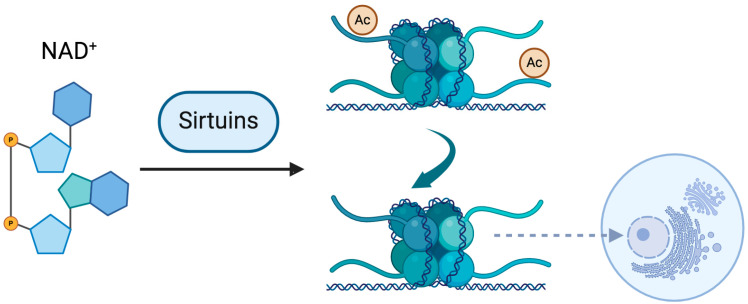
Sirtuins are NAD-dependent enzymes that function as histone deacetylases (HDACs), playing a pivotal role in chromatin regulation. The deacetylation of histone proteins is associated with nucleosome displacement and the relaxation of chromatin structure. The nucleolus, a region of pronounced chromatin compaction, requires this structural integrity to preserve genomic stability. The proximity of sirtuins to the nucleolar domain has been recognized as a crucial determinant in safeguarding nucleolar chromatin stability. Created in BioRender. Souza, A. (2025) https://BioRender.com/bunnp4l (accessed on 21 March 2025).

Sirtuins, as NAD^+^-dependent histone deacetylases (HDACs), are instrumental in maintaining chromatin organization and genomic stability, with a particularly crucial role in the structural integrity of the nucleolus [68,69]. The nucleolus, a specialized nuclear domain dedicated to ribosomal RNA (rRNA) synthesis and ribosome biogenesis, undergoes dynamic remodeling in response to cellular stress and aging. SIRT7, a nucleolar sirtuin, has been shown to regulate nucleolar chromatin condensation by deacetylating histone H3 on lysine 18 (H3K18Ac), a modification associated with transcriptional repression and genomic stability [70]. This activity is essential for preserving nucleolar architecture and preventing rDNA instability, a hallmark of cellular aging and tumorigenesis. Additionally, SIRT1 and SIRT6 contribute to nucleolar homeostasis by repressing excessive rDNA transcription, thereby mitigating hyperactive ribosome biogenesis, which is frequently associated with pro-aging phenotypes and oncogenic transformation [71,72]. Notably, polyphenols such as resveratrol and quercetin, through their ability to activate sirtuins, may exert protective effects on nucleolar integrity by preserving rDNA stability and attenuating age-associated chromatin deregulation [6,58,59]. These findings position the nucleolus as a central hub in the interplay between epigenetics, aging, and polyphenol-mediated healthspan extension (Figure 1).

Emerging evidence suggests that polyphenols act as potent hormetic agents, eliciting adaptive cellular stress responses that enhance metabolic homeostasis, genomic integrity, and longevity. Their pleiotropic effects extend well beyond direct antioxidant activity, encompassing metabolic reprogramming, autophagic flux modulation, and the maintenance of proteostasis (Table 1). As such, dietary polyphenols represent promising candidates for epigenetic-based therapeutic strategies aimed at counteracting the deleterious effects of aging and extending healthspan. However, challenges related to their bioavailability, metabolic transformation, and interindividual variability remain areas of active investigation, necessitating further translational research to harness their full potential in precision medicine and longevity science.

## 8. Astaxanthin: A Multifaceted Carotenoid with Emerging Epigenetic Influence

The oil-soluble compound astaxanthin represents a potent red xanthophyll carotenoid naturally produced by the microalga *Haematococcus pluvialis* and *Chlamydomonas nivalis*, under environmental stress, as well as in crustaceans such as fish, shrimp, and fish eggs, yeast, bacteria, and plants. Astaxanthin exhibits remarkable antioxidant and anti-inflammatory properties, significantly contributing to the improvement of cellular redox status. Its unique molecular structure, featuring two carbonyl groups, two hydroxyl groups, and eleven conjugated double bonds, underpins its superior capacity to neutralize reactive oxygen species (ROS), including singlet oxygen, and inhibit lipid peroxidation. Scientific evidence consistently demonstrates astaxanthin’s efficacy in mitigating oxidative stress by reducing markers such as malondialdehyde (MDA) and enhancing the activity of endogenous antioxidant enzymes like superoxide dismutase (SOD), particularly evident in clinical studies involving conditions like type 2 diabetes mellitus [85,86].

Furthermore, astaxanthin exerts powerful anti-inflammatory effects through the modulation of key molecular pathways, notably the suppression of NF-κB activation, leading to a reduction in pro-inflammatory cytokines such as interleukin-6 (IL-6). This dual action—robust antioxidant activity coupled with potent anti-inflammatory modulation—collectively contributes to the preservation and improvement of cellular redox balance, offering substantial protective benefits against oxidative damage and inflammation-driven pathologies across various physiological systems [86,87].

Recent scientific inquiry highlights its capacity to affect processes such as microRNA (miRNA) expression, which are crucial regulators of post-transcriptional gene silencing. Studies suggest that astaxanthin supplementation can modulate specific miRNA profiles, consequently impacting pathways linked to inflammation and metabolic health [87,88]. While research on direct impacts on DNA methylation and histone modifications by astaxanthin is still nascent, its ability to mitigate oxidative stress and inflammation creates an environment conducive to maintaining healthy epigenetic patterns. This expanding understanding points to astaxanthin’s broader influence on cellular function and disease prevention through epigenetic regulation.

## 9. Conclusions

The diet serves as a crucial source of nutrients and bioactive molecules essential for the optimal operation of cellular metabolic biochemical pathways. Notably, some of these pathways are significant generators of substrates, co-substrates, and cofactors for enzymes that indicate the epigenetic landscape of the cell genome. An unbalanced diet that does not provide, either in quality or quantity, the requisite bioactive molecules for the maintenance of biochemical cycle homeostasis not only compromises cellular metabolic function but also perturbs gene transcription due to the consequent disruption promoted by epigenetic regulation. Considering that the synthesis of all proteins and enzymes taking part in the biochemical pathways of cellular metabolism is also affected by epigenetic deregulation, it is clearly understood that it is a bidirectional and self-reinforcing pathway that can generate deleterious outcomes when unbalanced.

## Figures and Tables

**Table 1:** Dietary sources of polyphenols and their roles in cellular metabolism and epigenetic regulation.

Class	Polyphenol	Diet Source	Function in Metabolism and Epigenetics	References
Flavonoid	Quercetin	Apples, onions, capers, berries	Quercetin has been implicated in the regulation of DNA methylation, influencing various gene expression profiles linked to inflammatory and cellular stress responses. This flavonoid also exhibits potential to modulate epigenetic enzymes, promoting a favorable genomic environment.	[73]
Flavonoid	Kaempferol	Kale, spinach, broccoli, leeks, beans	This flavonoid is known to induce significant changes in DNA methylation patterns and histone acetylation, thus altering the expression of genes involved in cell differentiation and apoptosis, with implications for cancer prevention.	[74]
Flavonoid	Catechins	Green tea, black tea, dark chocolate	Catechins have been shown to modulate various histone modifications, particularly enhancing acetylation, which can lead to the upregulation of genes associated with antioxidant activity and downregulation of pro-inflammatory pathways.	[75,76]
Flavonoid	Anthocyanins	Berries (blueberries, blackberries), red cabbage, eggplant	These potent antioxidants are thought to induce epigenetic modifications that can counteract oxidative stress and inflammation, facilitating a protective response through modulation of gene expression related to cellular health.	[77]
Flavonoid	Hesperidin	Citrus fruits (oranges, lemons), peppermint, berries	Hesperidin plays a significant role in modulating gene expression related to oxidative stress response, potentially influencing signaling pathways crucial for maintaining cellular homeostasis through epigenetic mechanisms.	[78]
Non-Flavonoid	Resveratrol	Red wine, grapes, peanuts, berries	Resveratrol exhibits a remarkable capacity to influence histone acetylation, which in turn regulates the expression of genes involved in longevity and metabolic health, highlighting its potential in disease prevention strategies.	[79]
Non-Flavonoid	Curcumin	Turmeric, ginger, curry powder, mustard	Known for its multifaceted actions, curcumin can trigger significant epigenetic alterations in histone modification, impacting the expression of genes associated with inflammation, cancer progression, and neuroprotection.	[80]
Non-Flavonoid	Oleuropein	Extra virgin olive oil, olives, olive leaf extract	This polyphenol is recognized for its role in modulating key epigenetic factors involved in metabolic pathways, potentially influencing lipid metabolism and inflammatory responses, thus promoting overall health.	[81]
Non-Flavonoid	Gallic acid	Black tea, walnuts, grapes, pomegranates	Gallic acid is believed to affect DNA methylation dynamics, particularly in genes related to immune responses and cancer susceptibility, promoting a balanced epigenetic landscape.	[82,83]
Non-Flavonoid	Anthocyanidins	Grapes, apples, cocoa, berries	These compounds exhibit the capacity to influence epigenetic regulation concerning antioxidant defense systems, thereby mediating protective effects against oxidative damage and chronic diseases.	[84]

## Abbreviations

The following abbreviations are used in this manuscript:

3′ UTR	3′ untranslated regions
5,10-MTHF	5,10-methylenetetrahydrofolate
ACC1	Acetyl-coa Carboxylase 1
Acetyl-CoA	Acetyl-coenzyme a
ALKBH5	Alkb homolog 5
AMPK	AMP-activated Protein Kinase
ARE	Antioxidant response element
ATP	Adenosine triphosphate
BHMT	Betaine-homocysteine methyltransferase
CBP	CREB-binding protein
CBS	Cystathionine β-synthase
CpG	Cytosine-phosphate-Guanine
DNMT	DNA methyltransferase
dTMP	Deoxythymidine 5′-monophosphate
dUMP	Deoxyuridine 5′-monophosphate
EGCG	Epigallocatechin-3-gallate
FADH2	Flavin adenine dinucleotide
FOXO	Forkhead box O
FTO	Fat mass and obesity-associated protein
H3K18Ac	Histone h3 lysine 18 acetylation
HAT	Histone acetyltransferase
HCY	Homocysteine
HDAC	Histone deacetylase
HMT	Histone methyltransferases
JHDM	Jumonji C-domain-containing histone demethylases
LINE-1	Long interspersed nuclear element-1
m6A	N6-methyladenosine
METTL	Methyltransferase-like
MS	Methionine synthase
MTHFR	Methylenetetrahydrofolate reductase
mTOR	Mechanistic Target of Rapamycin
NAD^+^	Nicotinamide adenine dinucleotide
NADH	Nicotinamide adenine dinucleotide
ncRNAs	Non-coding rnas
NF-kB	Nuclear factor kappa b
NMN	Nicotinamide mononucleotide
Nrf2	Nuclear factor erythroid 2-related factor 2
rDNA	Ribosomal DNA
ROS	Reactive oxygen species
rRNA	Ribosomal RNA
SAH	S-adenosylhomocysteine
SAM	S-adenosylmethionine
SIRT	Sirtuin
SNP	Single nucleotide polymorphism
TCA	Tricarboxylic acid cycle
TET	Ten-eleven Translocation
